# Tackling Issues Observed during the Development of a Liquid Chromatography Method for Small Molecule Quantification in Antibody-Chelator Conjugate

**DOI:** 10.3390/molecules28062626

**Published:** 2023-03-14

**Authors:** Thomas Bouvarel, Nadine Bremeyer, Mimi Gao, Wiebke Holkenjans, Terence Hetzel, Reinhard Pell, Valentina D’Atri, Davy Guillarme

**Affiliations:** 1 School of Pharmaceutical Sciences, University of Geneva, CMU—Rue Michel Servet 1, 1211 Geneva, Switzerland; 2 Institute of Pharmaceutical Sciences of Western Switzerland, University of Geneva, CMU—Rue Michel Servet 1, 1211 Geneva, Switzerland; 3 Bayer AG, 42096 Wuppertal, Germany

**Keywords:** antibody-chelator conjugate, radionuclide, reversed-phase liquid chromatography, thorium-227 conjugate

## Abstract

In the context of targeted radionuclide therapy, antibody-chelator conjugates (ACCs) are an evolving class of antibody-related drugs with promising applications as tumor-targeted pharmaceuticals. Generally, a typical ACC consists of a recombinant monoclonal antibody (mAb) coupled to radionuclide via a chelating agent. Characterizing the ACC structure represents an analytical challenge since various impurities must be constantly monitored in the presence of formulation components during the quality control (QC) process. In this contribution, a reliable method devoted to the monitoring of an ACC sample, and its small molecule-related synthesis impurities, has been developed via liquid chromatography (LC). A problem-solving approach of common analytical issues was used to highlight some major issues encountered during method development. This included separation of poorly retained impurities (issue #1); interferences from the formulation components (issue #2); analysis of impurities in presence of ACC at high concentration (issue #3); and recovery of impurities during the whole analytical procedure (issue #4). To the best of our knowledge, this is the first time that a chromatographic method for the analysis of ACC synthesis impurities is presented. In addition, the developed approach has the potential to be more widely applied to the characterization of similar ACCs and other antibody-related drugs.

## 1. Introduction

The importance of monoclonal antibodies (mAbs) is continuously increasing, both in terms of sales and their applications for the treatment of a wide range of clinical indications, such as cancer and autoimmune diseases [1]. Therapeutic mAbs are often described as the fastest-growing class of medicines in recent decades, with 12 new products granted first approvals by the United States Food and Drug Administration (FDA) and the European Medicines Agency (EMA) in 2022 and 4 mAb products forecasted to be in the top ten selling drug products in 2021 [2,3]. In total, more than 100 mAbs have already been approved by the FDA or the EMA [4]. The long-term outlook also looks promising, as the commercial pipeline of 2022 includes more than 800 antibody therapeutics in clinical development [2]. The success of these highly efficacious therapies is largely fueled by the intrinsic characteristics of mAbs that provide enhanced target specificity and unique pharmacokinetic and pharmacodynamic (PK/PD) properties, compared to traditional small molecular drugs [5] Based on the therapeutic and commercial success of mAbs, many new antibody-based drug formats have emerged over the past decade, with ever-increasing structural complexity and therapeutic applications [2,6]. These include bi-specific antibodies (bsAbs), peptibodies, Fc-fusion proteins, and antibody-drug conjugates (ADCs), as well as antibody-chelator conjugates (ACCs). ACCs are cell-targeting recombinant mAbs designed to couple radionuclides via a chelator agent [7]. They could be employed to visualize the in vivo distribution of target cell surface receptors, in combination with an imaging probe [7,8]. However, if radionuclides are either alpha, beta and/or gamma radiation emitters, then ACCs are not only valuable as imaging agents. They can be additionally explored as potential platforms for specific tumor-targeted pharmaceuticals [8,9,10,11,12]. It is therefore possible to exploit the in vivo vector properties of mAbs, to deliver an additional radionuclide payload and take advantage of the targeted killing power of these isotopes on tumor cells with minimized damage to surrounding healthy tissue. As example, one-third of the radiopharmaceuticals in clinical trials in 2021 for prostate cancer involved mAbs as viable radionuclide delivery vehicles [13].

**Figure 1:**
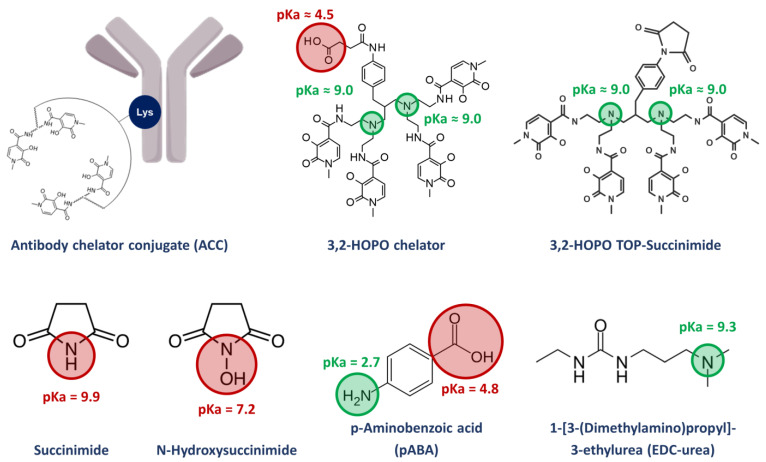
Synthesis impurities and stabilizer of the ACC with their respective p*K_a_* values.

When designing an endoradionuclide therapy, the bifunctional chelating agent, coordination of the radionuclide and conjugation to the targeting moiety play a crucial role: since radionuclides are constantly decaying and biological macromolecules such as mAb feature limited thermal stabilities, a chelator allowing for rapid and convenient complexation in a near quantitative yield and under mild conditions is advantageous [14,15,16]. Recently, a very efficient and selective octadentate chelator for thorium-227 complexation has been developed [17,18,19]. It is based on a symmetrical polyamine scaffold bearing four 3-hydroxy-*N*-methyl-2-pyridinone (3,2-HOPO) moieties and functionalized with a reactive carboxylic acid linker for bioconjugation (Figure 1). Upon activation in the form of an active *N*-hydroxysuccinimide ester, the chelator can be readily conjugated to a wide range of targeting moieties, i.e., mAb through the amide bond formation with the ɛ-amino groups of lysine residues [17,18,19]. Kinetic experiments demonstrated the labeling of the bioconjugated chelator with thorium-227 to be completed within 1 h at room temperature, enabling the generation of new targeted thorium-227 conjugates (TTCs) [12,20]. With a half-life of 18.7 d, TTC drug products have an extremely limited shelf life, requiring a front-loaded quality control (QC) strategy with extensive testing on the ACC level before the addition of the radionuclide and limited testing of the final drug product (hot material). From an analytical point of view, the detailed characterization of the impurity profile of ACCs could be difficult, time-consuming, and require the use of powerful chromatographic methods. Some strategies for the analytical characterization of ACCs have been reported in the literature, such as the calculation of the chelator to antibody ratio (CAR) and using size exclusion chromatography (SEC) methods [21,22,23]. The dimerization and aggregation of an ACC for the treatment of mesothelioma and ovarian cancer was also assessed via SEC [24]. At the hot material level, the radiochemical purity (RCP), defined as the ratio of bound to free metal radionuclide fraction, is a critical quality attribute (CQA) [21,24]. Another important TTC-specific CQA is the residual level of free chelators and other conjugation process-related small molecules. The manufacturing and work-up process aims for the removal of such impurities on the ACC level. However, due to their off-targeted genotoxic potential when labelled with thorium-227, potential residual amounts be analytically monitored and tightly controlled. The small molecule impurities of interest can feature very diverse physico-chemical properties and complicate the analytical work in terms of chromatographic separation. The usage of one single stationary phase for the simultaneous separation and quantification of all relevant small molecule impurities is desired but currently not reported in the literature. To the best of our knowledge, the methods developed to characterize two of the impurities that will be investigated in this work, namely *N*-hydroxysuccinimide (NHS), and succinimide, are quite rare, complicated, and mostly indirect. Only one paper from 2015 reports a HILIC method for the successful determination of NHS [25]. On the other hand, we do not find methods for the other impurities found in ACC products (3,2-HOPO chelator and its by-product 3,2-HOPO TOP-succinimide) as well as the process related impurities 1-[3-(dimethylamino)propyl]-3-ethylurea (EDC-urea).

In this work, we describe the development of a unique reverse phase liquid chromatography (RPLC)-UV-MS method that can separate and quantify all relevant small molecules derived from an NHS ester-assisted bioconjugation of the 3,2-HOPO chelator, even in a complex formulation of the ACC. As shown in Figure 1, this included the two free chelators 3,2-HOPO chelator and its by-product 3,2-HOPO TOP-succinimide, as well as the process related impurities *N*-hydroxysuccinimide (NHS), succinimide and 1-[3-(dimethylamino)propyl]-3-ethylurea (EDC-urea) from the EDC/NHS-mediated bioconjugation. To develop a reliable analytical method, multiple constraints and challenges were accounted. The retention of the early eluting compounds in RPLC, i.e., succinimide and *N*-hydroxysuccinimide, was first investigated by screening a large panel of stationary phases having a significant polar surface activity followed by further refinement in terms of column temperature and mobile phase pH. The later was investigated to avoid possible interferences from the excipients of the ACC formulation, required to ensure the solubility and stability of the chelator. In addition, the analysis of impurities was performed in presence of ACC at high concentration by implementing a filtration process. Finally, the validity of the analytical approach was also evaluated.

## 2. Results and Discussion

As illustrated in Figure 1, the process related impurities that can be found in the ACC product are quite diverse in terms of physico-chemical properties like p*K_a_* and log*P*. Therefore, special care was taken to develop a suitable method able to analyze all these low molecular weight species within a single analytical method. This article summarizes some of the issues we faced during method development, and which approaches were selected to overcome these challenges. Obviously, the approaches listed here can be helpful for the development of analytical methods of other ACC products having a similar chemistry.

The analytical target profile (ATP) is a useful tool to define a priori quality criteria for results generated by analytical methods. For the ACC studied in this work, the method criteria described below are linked to product specifications based on process capability. In the present work, the following criteria were defined: (i) the retention factor, *k*, should be at least equal to 1 for all peaks of interest; (ii) the overall chromatographic resolution should be at least equal to 1.5; (iii) the limit of quantitation (LOQ) should be ideally equal to or below 0.1 µg/mL for EDC-urea, 4 µg/mL for succinimide and *N*-hydroxysuccinimide, and 0.05 µg/mL for 3,2-HOPO-chelator and 3,2-HOPO-TOP-succinimide; (iv) linearity should be not less than R^2^ = 0.998; and (v) precision should be less than 2% and 6% for UV and MS detection, respectively. Each of these attributes will be measured and compared with expected values.

### 2.1. Issue #1: Retention and Detection of Succinimide and N-hydroxysuccinimide

In terms of physico-chemical properties, succinimide has a log*P* of −0.99 and an acidic p*K_a_* of 9.9, while *N*-hydroxysuccinimide has a log*P* of −0.99 and an acidic p*K_a_* of 7.2. It is well known that RPLC mode is suited for the analysis of compounds having log*P* ranging from −1 to 6. However, due to the relatively low and very similar log*P* values of succinimide and *N*-hydroxysuccinimide, these compounds may not be sufficiently and selectively retained on a regular C18 column (see issue #2 highlighting the potential problems encountered with selectivity). Therefore, alternative column chemistry allowing a highly aqueous mobile phase will be required to achieve a sufficient retention factor (ideally *k* at least equal to 1, as indicated in the ATP). Ion-pair-RPLC as a possible alternative is hardly applicable, due to the very low ionic character of the compounds at acidic, neutral, or slightly basic conditions. The ionization of these compounds would require the use of highly basic mobile phase conditions, which is hardly compatible with most of the C18 material on the market. Besides the limited retention in RPLC mode, it is also important to notice that succinimide and *N*-hydroxysuccinimide do not significantly absorb in UV above 200 nm, so this very low wavelength was selected for their detection.

First, it is important to note that no RPLC method is available in the literature to simultaneously analyze succinimide and *N*-hydroxysuccinimide. In the present work, rather than using a regular C18 column, we decided to test various commercially available RPLC stationary phases having a significant polar surface activity (see Supplementary Materials: Table S1 for the detailed characteristics of the columns) [26]. Among the tested columns, there were a fluorophenyl (CSH fluorophenyl), an ether linked phenyl phase (Synergi Polar-RP), a mixed-mode anion-exchange/RPLC column (Premier BEH C18 AX), and five C18 columns with polar endcapping from different providers (Premier HSS T3, Luna Omega polar, Hypersil GOLD aQ, Zorbax SB Aq, and Kinetex Polar). All the tested stationary phases had the same length, but the internal diameter was larger for the Luna Omega Polar and the Kinetex polar (3 mm I.D.), compared to the other ones (2.0–2.1 mm I.D.). Thus, the flow rate was geometrically adjusted at 0.8 mL min^−1^ for the 3 mm I.D. columns and at 0.4 mL min^−1^ for the 2.0 and 2.1 mm I.D. ones. In terms of particle morphology, most of the columns were packed with fully porous particles ranging from 1.7 to 3.0 µm, while the Kinetex polar was packed with superficially porous particles of 2.6 µm. In all cases, the column void time varied from 0.60 to 0.89 min and was obtained from the baseline disturbance observed at 200 nm in UV. All of these columns were tested under generic mobile phase conditions and further improvements were then brought to the method afterwards.

The analysis of succinimide and *N*-hydroxysuccinimide was carried out using purely aqueous acidified mobile phase to maximize retention and achieve sufficient selectivity between excipients and impurities (see case study #2). Obviously, not all RPLC columns can be used under such conditions, due to the well-known phase dewetting phenomenon [27]. Phase dewetting is highly undesirable; since retention times decrease and are not reproducible, peaks may become distorted and re-equilibration times may be quite long [28,29]. To avoid such issue, a phase that is solvated under all mobile phase conditions has to be preferentially used (polar embedded phases, endcapping with a polar functionality, polar bonding…), such as the ones that were selected in this work.

Under these purely aqueous conditions, the Kinetex Polar provided suitable peak shapes and sufficient selectivity for succinimide and *N*-hydroxysuccinimide but was the less retentive stationary phase (*k* < 1 for both molecules). However, it is hard to interpret this result, as the polar endcapping employed for this stationary phase is proprietary and no information is available from the provider. The CSH fluorophenyl was also poorly retentive (*k* < 1) and provided unsuitable peaks shapes (strong tailing) and limited selectivity. This phase possesses a positively charged surface (along with the fluorophenyl group). It should provide improved peak shapes and weaker retention for bases, while acids should be more strongly retained through the ion exchange mechanism. However, in the present case, both succinimide and *N*-hydroxysuccinimide were under their neutral form at the selected pH (~2), and, therefore, the positively charged surface did not increase retention of these two molecules. In addition, the charge transfer retention mechanism produced by the fluorinated ligand was found to be unsatisfactory to retain succinimide and *N*-hydroxysuccinimide [30].

A very similar chromatographic behavior was observed with the Hypersil GOLD aQ and Zorbax SB Aq columns. Retention was better compared to the previously described stationary phases, but still insufficient. Here, again, no information is available from the provider regarding the nature of the polar ligand employed for polar endcapping. The Premier BEH C18 AX column, a mixed-mode stationary phase specifically developed to retain polar acidic analytes, was also found to be insufficiently retentive, mostly due to the fact that the targeted compounds were neutral under the selected mobile phase conditions [31].

**Figure 2:**
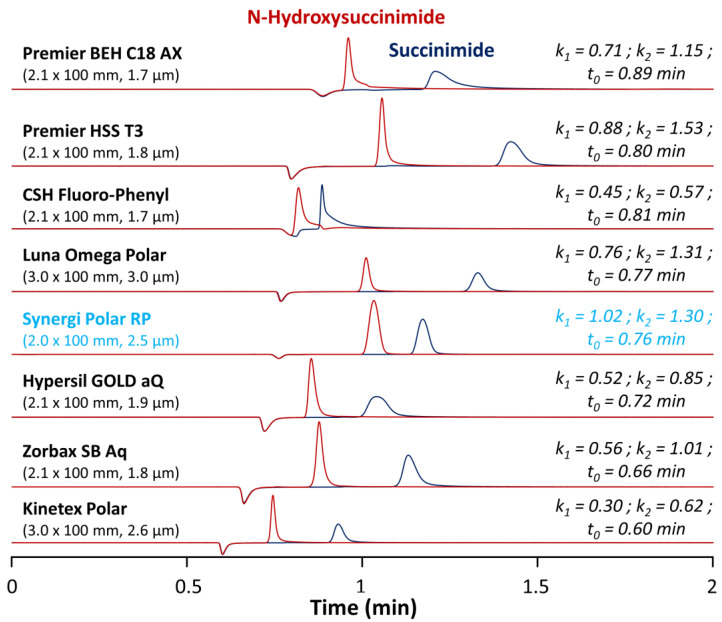
Chromatograms illustrating the screening results for separation of *N*-hydroxysuccinimide and succinimide obtained on eight different columns in isocratic mode with 0.1% TFA in water at 40 °C.

As shown in Figure 2, the most promising columns to analyze succinimide and *N*-hydroxysuccinimide were the Premier HSS T3, Luna Omega Polar, and Synergi Polar RP columns. These three columns offered suitable peak shapes, retention for the first eluted peak (*N*-hydroxysuccinimide) with retention factors *k* between 0.71 and 1.02, and a baseline separation of the two analytes. The good retention of polar analytes on the HSS T3 was due to the low ligand density (only 1.6 µmol/m^2^ vs. >3.0 µmol/m^2^ for regular C18 material) promoting the polar interaction with polar endcapped groups at the surface of the stationary phase. For the Luna Omega Polar stationary phase, the retention of succinimide and *N*-hydroxysuccinimide was exclusively due to the presence of a proprietary polar endcapping able to interact with these hydrophilic analytes. Among these three columns, the Synergi Polar RP was selected, as it was the only one offering retention factors *k* higher than 1 for the two compounds (this was a critical parameter considering the numerous components of the ACC formulation (as illustrated in case study #2) and the poorly selective detection wavelength of 200 nm). This stationary phase has multiple possibilities to interact with the two analytes of interest, thanks to its endcapping (proprietary polar ligand), the presence of a phenyl group, and ether linkage as the polar embedded group, resulting in improved peak shapes and suitable retention of acidic polar analytes.

### 2.2. Issue #2: Interferences from the Formulation Components

Since a low wavelength of 200 nm was selected to detect succinimide and *N*-hydroxysuccinimide, and as the retention of these molecules was limited, it was important to consider the possible interferences from some of the components of the ACC formulation (citric acid, sucrose, ethylenediaminetetraacetic acid (EDTA), and *p*-aminobenzoic acid (pABA)). Based on their UV spectra, all these additives are detectable at 200 nm. Therefore, it was necessary to ensure that they did not interfere with the early eluting analytes (succinimide and *N*-hydroxysuccinimide).

To evaluate whether the selectivity between excipients and analytes of interest is sufficient, all excipients were individually injected using the previously selected column (Synergi Polar RP). Importantly, a generic gradient was performed in the presence of 0.1% TFA, as it was found that TFA was critical to obtain sharp peaks for 3,2-HOPO chelator and its derivative 3,2-HOPO TOP-succinimide, due to the presence of two tertiary amines with a p*K_a_* of 9.0 in these two molecules. The gradient started with an isocratic step at 0% MeOH for 5 min to sufficiently retain succinimide and *N*-hydroxysuccinimide. Then, the proportion of MeOH was increased from 0% to 100% over 5 min for the elution of EDC-urea. Next, a second isocratic step at 100% MeOH was added for 5 min, allowing for the elution of 3,2-HOPO chelator and its derivative 3,2-HOPO TOP-succinimide. Finally, a re-equilibration of the column at the initial gradient conditions (as described in Section 3.3) was performed. To analyze all the compounds of interest, various detection conditions were employed to reach the expected limits of detection, namely UV at 200 nm for succinimide and *N*-hydroxysuccinimide, UV at 330 nm for the two free chelator species, and MS in selected ion recording (SIR) mode for EDC-urea (m/z 174.1). As previously discussed, succinimide and *N*-hydroxysuccinimide were eluted during the initial isocratic step with a purely aqueous mobile phase, while EDC-urea was eluted close to the end of this isocratic step. On the other hand, the two free chelator species were found to be much more hydrophobic, and their elution took place during the final isocratic step at 100% MeOH.

**Figure 3:**
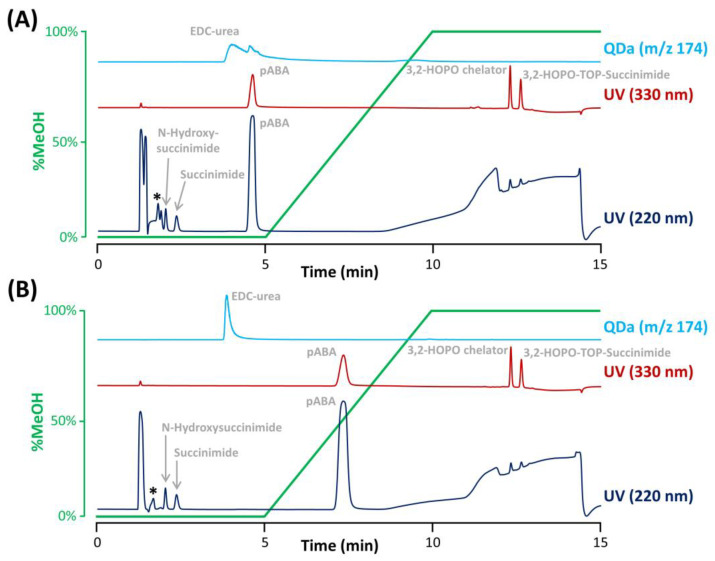
Separation of the ACC impurities and formulation components performed on the Synergi Polar RP column with an aqueous mobile phase containing (**A**) 0.1% TFA without pH adjustment or (**B**) 0.075%TFA and pH adjustment at 2.6 with ammonium hydroxide. An asterisk denotes the elution of citric acid and EDTA. The gradient profile was reported in green.

Under the selected conditions, most of the excipients were not problematic as they were either excluded from the pores of the column (elution before the baseline disturbance corresponding to *t*_0_), not retained (eluted at *t*_0_), or hardly detectable in UV at a wavelength of 200 nm at the concentration level of the formulation. However, the radiostabilizer pABA was retained on the Synergi Polar RP column due to the presence of an aromatic ring that can interact with the ether-phenyl bonding at the surface of the stationary phase, and eluted slightly before 5 min, far from the elution zone of succinimide and *N*-hydroxysuccinimide (see Figure 3A). However, pABA was eluted in the tail of the EDC-urea peak. Even if the SIR of EDC-urea was selective (m/z 174.1), pABA possesses an aniline group which can be ionized in the positive ESI mode. Therefore, we observed a competition for ionization between EDC-urea and pABA in the electrospray ionization source (matrix effects), leading to peak deformation observed in Figure 3A and inaccurate quantitation. 

Additionally, citric acid and EDTA were observed to be slightly retained under the selected conditions and eluted just before the peak of *N*-hydroxysuccinimide. An interesting solution to improve the separation of citric acid, EDTA, and *N*-hydroxysuccinimide was to tune the amount of TFA and mobile phase pH. Indeed, EDTA is a poly-acid with four different carboxylic acid groups having p*K_a_* of 0, 1.5, 2.0, and 2.66, and citric acid is a triprotic acid with p*K_a_* of 3.1, 4.7, and 6.4. On the other hand, *N*-hydroxysuccinimide has a significantly higher acidic p*K_a_* of 7.2. Based on these acid-basic properties, a slight increase of the mobile phase pH should provide a retention decrease for EDTA and citric acid as conditions are created so that the second and third protons can be donated, while the retention of *N*-hydroxysuccinimide should not be affected as its p*K_a_* is too far from the mobile phase pH of 2.0. In a first instance, different mobile phases differing in the proportion of TFA (i.e., 0.15%, 0.1%, 0.075%, and 0.03% TFA) were evaluated (data not shown). It appeared that the impact of this change was quite limited, mostly due to a narrow change in mobile phase pH produced. Nevertheless, the resolution between EDTA and *N*-hydroxysuccinimide (the two most critical compounds) was improved when using the aqueous mobile phase with 0.075% TFA resulting in a mobile phase pH of approx. 2.15. Therefore, this TFA proportion was kept for the rest of the study. To further improve selectivity between *N*-hydroxysuccinimide and the two interfering substances (citric acid and EDTA), the mobile phase was further adjusted to various pH comprised between 2.15 and 3.0, by adding ammonium hydroxide. Finally, the best separation was obtained when fixing the pH at 2.6, as illustrated in Figure 3B, where succinimide and *N*-hydroxysuccinimide were clearly separated from the interfering compounds.

In addition, the pH increase also impacted the elution of pABA, which possess a basic p*K_a_* of 2.7. pABA was therefore more retained at pH 2.6 (Figure 3B) vs. pH 2.0 (Figure 3A), while EDC-urea was not affected by this pH change (basic p*K_a_* of 9.3), overcoming the interference effects in MS detection. Interestingly, the peak shape of EDC-urea was also drastically improved due to the presence of ammonium hydroxide in the mobile phase (ammonium ions can mask residual silanols at the column surface, reducing undesirable secondary ionic interactions).

### 2.3. Issue #3: Analysis of Impurities in Presence of ACC at High Concentration

**Figure 4:**
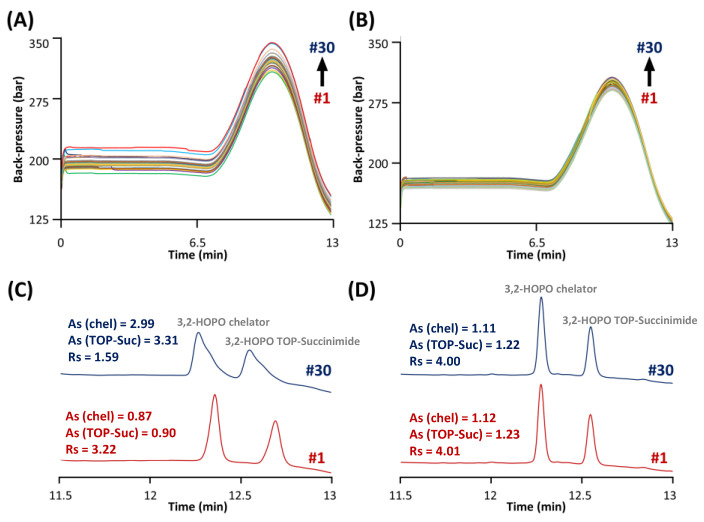
Evolution of the column back-pressure during 30 injections of ACC samples spiked with the synthesis impurities. Samples analyzed (**A**) before and (**B**) after the ACC removal through filtration. Chromatographic behavior of 3,2-HOPO chelator and its by-product TOP-succinimide by comparing the first (red) and 30th (blue) injection of samples analyzed (**C**) before and (**D**) after the ACC removal.

An additional constraint that was considered during the development of this method was the presence of ACC at high concentration. Indeed, it was necessary to inject the ACC product at 6 mg mL^−1^ to reach sufficient LOD/LOQ for the impurities. The stationary phase selected to analyse the impurities was obviously ideally suited for the analysis of ACC (molecular weight in the range of 150 kDa, hydrodynamic radius around 5–7 nm), as the pore size was extremely small (80 Å) [32]. As shown in Figure 4A, an increase in column backpressure by about 50 bar after only 30 injections of ACC at 6 mg mL^−1^ was observed. More importantly, the chromatographic performance was also strongly reduced, as shown in Figure 4C.

Indeed, the resolution between the two free chelator species was equal to 3.22 for injection #1, while this value drops to only 1.59 for injection #30. One of the reasons for this critical behaviour could be that impurities were analysed under RPLC conditions at only 20 °C in order to achieve sufficient retention for the early eluted impurities (i.e., succinimide and *N*-hydroxysuccinimide). However, it has been reported that antibody-based drugs such as monoclonal antibodies (mAbs) and antibody–drug conjugates (ADCs) can be strongly adsorbed at the surface of the stationary phase (mostly through ionic interactions and hydrogen bonding) at such a low temperature [33,34]. Since the ACC is an antibody-based drug, the same behaviour can be expected. Therefore, it can be stated that the accumulation of ACC (from the injections at high concentration) at the surface of the stationary phase can cause both a chemical modification of the Synergi Polar RP material (responsible for distorted peaks and modification of retention) and physical clogging of the stationary phase pores (responsible for pressure increase).

Unfortunately, it was not possible to solve this issue by simply increasing the column temperature in the range 70–90 °C, as the column is only stable up to 60 °C and the retention of succinimide and *N*-hydroxysuccinimide would be unacceptably low at the elevated temperature. An alternative solution that was tested in this work was ACC removal through filtration. Spin filters (Vivaspin) with a cut-off of 50 kDa were therefore used before the analysis by performing a centrifugation step at 10,000× *g* for 10 min at 20 °C. As shown in Figure 4B, an increase in column backpressure lower than 10 bar after 30 injections was observed after removal of the ACC. Figure 4D shows the separation of the two free chelator species at injections #1 and #30. As highlighted, the peak shapes, retention times, and chromatographic resolution of these two species were not affected after ACC removal.

### 2.4. Issue #4: Improvement of Recovery of Impurities

Being chelating agents, the 3,2-HOPO chelator and its by-product TOP-succinimide might easily capture or interact with metal ions, *e.g.*, iron(III), ubiquitously present in the analytical system [35,36]. Such interactions can cause deleterious effects ranging from peak tailing and generation of additional peaks to a complete loss of the analyte signal. To mitigate such interactions, we added the competitive chelator EDTA to the sample diluent and saturated the chelation sites by adding Th-232 (with a half-life of 14 billion years) in excess. As an additional care, we also systematically used polypropylene vials to limit the contact with metal ions, such as Na^+^ or K^+^. In our opinion, the addition of Th-232 to the samples was probably the most critical step to limit adsorption of 3,2-HOPO chelator and 3,2-HOPO TOP succinimide.

Besides the chelating properties of some impurities, the spin filter procedure for removal of large species (previously discussed) can also be a potential source of sample loss. In the present case, we used the classical polyethersulfone membrane recommended by the provider. By performing a semiquantitative evaluation of the method, which was obviously not intended to be a full method validation, we tried to understand whether impurities were lost during this step of the process and how to circumvent this issue.

**Figure 5:**
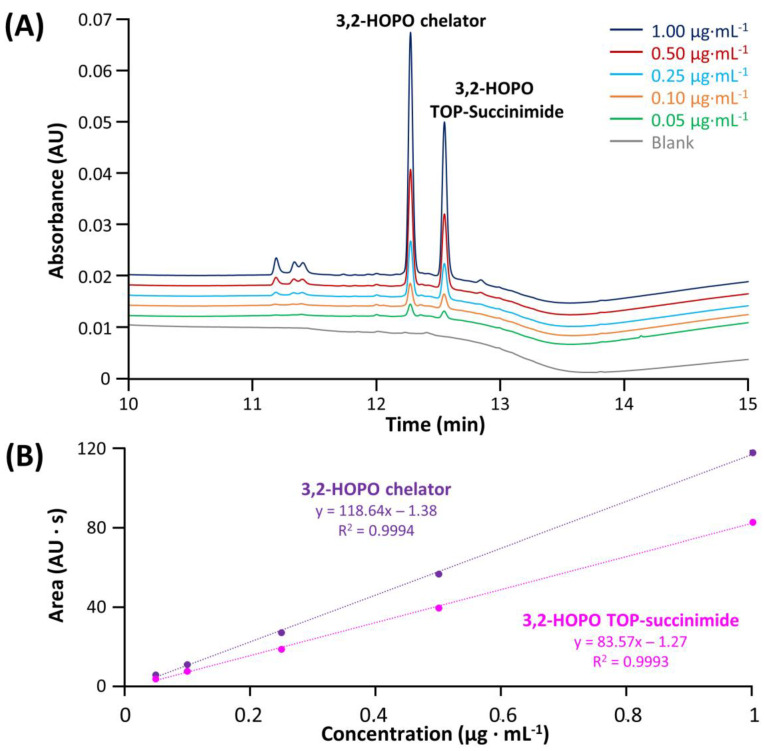
Chromatograms obtained after the filtration protocol for ACC samples (**A**) spiked with different concentrations of 3,2-HOPO chelator and 3,2-HOPO TOP-Succinimide and (**B**) resulting calibration lines with triplicate injections.

In a first instance, the method linearity and LOD/LOQ were evaluated for each impurity and compared with the ATP. The sample preparation protocol included the spiking of selected impurities in the ACC sample and then the filtration step performed with the Vivaspin. The corresponding LOD and LOQ corresponding to S/N ratio equal to 3 and 10, respectively, were summarized in Table S3. Figure 5 shows the chromatograms of 3,2-HOPO chelator and its by-product TOP succinimide at various concentrations ranging from 1 to 0.05 µg mL^−1^, in addition to a blank injection (grey trace). As shown from the blank injection, the specificity of the method was found to be given for these two compounds, and the compounds were easily detected at 0.05 µg mL^−1^, with resolution higher than 1.5. In addition, the regression lines were constructed. A linear model provides an *R*^2^ higher than 0.999 in both cases, with RSD values on peak area (n = 3) for the various concentrations ranging from 0.32–1.57% for 3,2-HOPO chelator and 0.43–1.36% for its by-product TOP succinimide, confirming the good linearity of the method in the investigated concentration range. Some injections of succinimide and *N*-hydroxysuccinimide at various concentrations comprised between 4 to 70 µg mL^−1^ were also carried out, and the corresponding data were reported in Figure S1. As illustrated, the linearity was higher than 0.998 for these two impurities and LOQ was well below the target level of 4 µg mL^−1^. Moreover, RSD values on area (n = 3) ranging from 0.18–0.49% and 0.08–0.42% were obtained for succinimide and *N*-hydroxysuccinimide, respectively. Last but not least, EDC-urea was injected at concentrations varying from 0.1 to 4 µg mL^−1^ and detected with MS detector operating in SIR mode (Figure S2). Here again, the method linearity was found to be excellent (R^2^ > 0.999), RSD values on area (n = 3) ranging from 0.64–1.56% were determined for the various concentrations, and the lowest concentration could be easily detected. All the LOD/LOQ and linearity were found to be in line with the ATP criteria.

Next, the recovery and precision of the method was checked for a sample containing all the impurities. For this purpose, the sample preparation protocol included the spiking of the five impurities in the ACC sample. Results obtained with and without the filtration step were first compared, and data were summarized in Table 1. In terms of precision (RSD on peak areas for three injections) for the sample without filtration, the values ranged from 0.22 to 0.89% for the four impurities detected in UV, while the RSD value was equal to 4.13% for the EDC-urea detected in MS. This behaviour is logical, as ESI-MS is known to produce much larger signal variability than UV detection. In the case where the sample was filtrated, the RSD values were slightly higher, comprised between 0.42 and 1.83%, while the RSD of EDC-urea was equal to 5.46%. From these results, it can be concluded that filtration does not alter the method precision, as comparable values were obtained with and without the filtration step. Thus, the precision of the method after filtration was found to be acceptable for a routine application of the method (<2% with UV detection and <6% with MS detection, in line with the ATP criteria). On the other hand, the average filtration recoveries (calculated by dividing the peak area without filtration by the peak area with filtration) were comprised between 89.3 and 91.3% for the first eluting peaks (*N*-hydroxysuccinimide, succinimide, and EDC-urea), while the filtration recoveries were equal to 59.7% and 68.4% for the 3,2-HOPO chelator and its by-product TOP succinimide, respectively. These two last molecules are the most retained in the chromatographic method (eluted with pure MeOH) and are therefore the most lipophilic ones. The sample loss could be due to the quite hydrophobic composition of the membrane (polyethersulfone membrane), which could generate some chemical interactions with the two chelator species [37]. Unfortunately, due to the large polarity range of the different impurities, the use of a more hydrophilic membrane does not seem to be a way to solve the problem for the lipophilic compounds, as it would most certainly result in a consequent loss of the more hydrophilic species.

As the 3,2-HOPO chelator and its by-product TOP succinimide were the most critical impurities for the filtration step, the method variability between six different injections from a single filtration and six injections from six independent filtrations were compared. The corresponding data were reported in Table 2.

As shown, the variability was slightly higher when performing independent filtrations, but remains acceptable, lower than 5%. Finally, the results reported in Table 1 and Table 2 prove that a non-negligible proportion of 3,2-HOPO chelator and its by-product TOP succinimide were lost during the filtration procedure (mostly due to the hydrophobic nature of the membrane). However, since the observed loss was constant between different filtration procedures (RSD values around 3%), it is recommended to apply the same strategy to the external calibrants and the unknown samples (namely adding a filtration step) to achieve suitable method trueness. In other words, using such a procedure, the developed method could be applied in a semiquantitative way to support in-process control activities.

## 3. Materials and Methods

### 3.1. Chemical and Reagents

Ultrapure water was obtained from a MilliQ purification system from Millipore (Bedford, MA, USA). Acetonitrile (ACN) and methanol (MeOH) were LC-MS grade (Optima^®^) and obtained from Fisher Chemical (Reinach, Switzerland). Trifluoroacetic acid (TFA, ≥99.0%) was obtained from Biosolve BV (Valkenswaard, the Netherlands). Thorium-232 (Th) pure standard at 1.0 mg mL^−1^ in 2% HNO_3_ was purchased from PerkinElmer (Schwerzenbach, Switzerland). Ammonium hydroxide (28.0–30.0%), citric acid (≥99.5%), ethylenediaminetetraacetic acid disodium salt dihydrate (EDTA, 99.0–101.0%), formic acid (≥98.0%), MES monohydrate (≥99.5%), *N*-(3-dimethylaminopropyl)-*N*′-ethylcarbodiimide hydrochloride (EDC, ≥98.0%), *N*-hydroxysuccinimide (≥99.0%), *N,N*-dimethylacetamide (DMA, 99.8%), *p*-aminobenzoic acid (pABA, 99.0%), and succinimide (≥98.0%) were obtained from Sigma-Aldrich (Buchs, Switzerland). The 3,2-HOPO chelator and TOP-succinimide, as well as the ACCs, were provided by Bayer AG (Wuppertal, Germany).

### 3.2. Instrumentation and Columns

Measurements were performed on Acquity UPLC I-Class system (Waters, Milford, MA, USA) equipped with a binary solvent delivery pump, an autosampler including a 15 µL flow-through-needle injector, and a photodiode array (PDA) detector (200 nm and 330 nm, 5 Hz), including a 500 nL low dispersion analytical flow-cell. In addition, the system was hyphenated to a Waters QDa single quadrupole mass spectrometer, fitted with a Z-spray ESI source. The MS device was operated in ESI positive mode with a capillary voltage of 0.8 kV, a desolvation temperature of 500 °C and a cone voltage of 15 V. Nitrogen (N_2_) was used as both desolvation and cone gas. All MS analyses were performed in ESI positive mode with a selected ion recorded (SIR) mass at m/z of 174.1 for EDC-urea. Data acquisition and instrument control were performed by Empower Pro 3 software.

In total, eight different columns were tested under RPLC conditions. The Acquity UPLC CSH fluorophenyl column (2.1 × 100 mm, 1.7 µm), the Premier BEH C18 AX column (2.1 × 100 mm, 1.7 µm), and the Premier HSS T3 column (2.1 × 100 mm, 1.8 µm) were purchased from Waters. The Luna Omega Polar C18 column (3.0 × 100 mm, 3.0 µm), the Kinetex Polar C18 column (3.0 × 100 mm, 2.6 µm), and the Synergi Polar RP column (2.0 × 100 mm, 2.5 µm) were obtained from Phenomenex (Torrance, CA, USA). The Hypersil GOLD aQ (2.1 × 100 mm, 1.9 µm) and the Zorbax SB Aq (2.1 × 100 mm, 1.8 µm) were purchased from Thermo Fisher Scientific (Reinach, Switzerland) and Agilent Technologies (Basel, Switzerland), respectively.

### 3.3. Chromatographic Conditions

The screening of RPLC columns was performed by investigating the separation of *N*-hydroxysuccinimide and succinimide with an isocratic mobile phase composed of 0.1% TFA in water. The flow rate was 400 µL min^−1^, column temperature was 40 °C and injection volume was 1 µL.

For the impurity profiling of ACC product, the Synergi Polar RP column (2.0 × 100 mm, 2.5 µm) was selected. Mobile phase A (MPA) was composed of water containing 0.075% TFA at pH 2.6 adjusted with ammonium hydroxide, while mobile phase B (MPB) was composed of MeOH containing 0.075% TFA. The injection volume was set at 5 μL, column temperature at 20 °C, and flow rate was set at 200 µL min^−1^. The gradient started with an initial isocratic step of 5.0 min at 0 %B. Then, the gradient increased from 0 to 100 %B in 5.0 min and remained at 100 %B for 5.0 min. Finally, the mobile phase composition was set back to 0 %B in 0.1 min to let the system equilibrating during 15 min. Pure ACN and a mixture of H_2_O/ACN (90/10, *v*/*v*) were used as strong and weak needle washes, respectively.

### 3.4. Sample Preparation

Prior to the preparation of the different samples required for this study, stock solutions of each individual analytes were prepared using an appropriate sample diluent. *N*-hydroxysuccinimide and succinimide were dissolved at 1.0 mg mL^−1^ in pure water. Stock solutions of 3,2-HOPO chelator and TOP-succinimide were prepared at 0.2 mg mL^−1^ in DMA/0.1 M MES buffer (50/50, *v*/*v*). To obtain a stock solution of EDC-urea at 0.2 mg mL^−1^, EDC was hydrolyzed at the same concentration in acidified water with 0.1% formic acid for 30 min at room temperature.

In the specific part of the work related to the column screening, a standard solution containing *N*-hydroxysuccinimide and succinimide at 0.4 mg mL^−1^ was prepared by diluting each corresponding stock solution in pure water and stored at 4 °C until further analysis.

For the optimization of the separation on the selected Synergi Polar RP column, a solution containing the five impurities at 5.0 µg mL^−1^ (for EDC-urea) and 10.0 µg mL^−1^ (for succinimide, *N*-hydroxysuccinimide, 3,2-HOPO chelator, and 3,2-HOPO TOP-Succinimide) was prepared via dilution with a solution containing 0.5 mg mL^−1^ pABA and 0.744 mg mL^−1^ EDTA in 30 mM citric acid at pH 5.5. A thorium-232 solution at 1.0 mg mL^−1^ was then immediately added to obtain a 210-fold molar excess of Thorium over the chelator and intermediate, to stabilize their chemical structures prone to oxidation and avoid the formation of other metal complexes.

Similarly, solutions of ACC at 6 mg mL^−1^ spiked with the five impurities at the different desired concentration levels were prepared. The same solution containing 0.5 mg mL^−1^ pABA and 0.744 mg mL^−1^ EDTA in 30 mM citric acid at pH 5.5 was used for dilution. The thorium solution at 1.0 mg mL^−1^ was immediately added to obtain a 7-fold molar excess over the ACC. The ACC was then removed using spin filters (Vivaspin 500, MWCO 50.0 kDa) from Sigma-Aldrich by centrifugating the sample at 10,000× *g* during 10 min at 20 °C. Final solutions were then transferred to HPLC vials for analysis.

## 4. Conclusions

With the advent of ACCs as new drug modalities, there is a growing need to support quality control (QC) with chromatographic methods that can properly monitor the ACC impurities. In this work, a single RPLC method was developed for the analysis of synthetic impurities of an ACC product. The investigated panel was composed of five impurities that could be found in the final ACC product, all having diverse physico-chemical properties and role in the synthetic pathway. Multiple analytical challenges were faced and solved to obtain a single RPLC method able to: (i) retain and separate poorly retained impurities (i.e., succinimide and N-hydroxysuccinimide); (ii) handle with possible interferences from the components of the ACC formulation (i.e., pABA, citric acid, and EDTA); (iv) cope with a high concentration of ACC; and (iv) obtain a robust method able to quantify the selected impurities (i.e., succinimide, N-hydroxysuccinimide, EDC-urea, 3,2-HOPO chelator, and TOP succinimide) in presence of the ACC at high concentration.

Selecting the most appropriate RPLC column, mobile phase and gradient conditions was crucial to obtain the best chromatographic performance. This included the Synergi Polar RP column used with mobile phase B (MPB) being 0.075% TFA in MeOH, and mobile phase A (MPA) 0.075% TFA in water, pH 2.6. In addition, implementing a filtration process to remove the ACC from the sample was mandatory to meet the sufficient LOD/LOQ for the impurities and achieve a sufficient degree of robustness, when injecting the ACC product at high concentrations (6 mg mL^−1^). Finally, the performance of the analytical method in terms of specificity, linearity, LOD/LOQ, and recovery were evaluated, and were found to be in line with the criteria defined in the ATP. To further improve the recoveries and RSD values, it could be possible to add an internal standard to the mixture before filtering and/or before MS analysis. However, this is not a simple task considering the chemical diversity of the analysed compounds.

To the best of our knowledge, this is the first time that a chromatographic method for the determination of impurities of an ACC is presented. In addition, the developed approach has the potential to be more widely applied to the characterization of similar ACCs and other antibody-related drugs such as ADCs.

## Figures and Tables

**Table 1:** Investigation of the filtration protocol effects for the 5 impurities spiked in an ACC sample (*N*-hydroxysuccinimide and Succinimide at 10 µg mL^−1^ + EDC-urea, 3,2-HOPO chelator, and TOP-succinimide at 0.5 µg mL^−1^ + ACC at 6 mg mL^−1^).

	RSD (Area) without Filtration (n = 3)	RSD (Area) with Filtration (n = 3)	Average Filtration Recovery (n = 3)
***N*-hydroxysuccinimide**	0.37%	0.42%	91.3%
**Succinimide**	0.22%	0.88%	88.5%
**EDC-urea**	4.13%	5.46%	89.3%
**3,2-HOPO chelator**	0.87%	1.83%	59.7%
**3,2-HOPO TOP-succinimide**	0.89%	1.66%	68.4%

**Table 2:** Repeatability of the filtration protocol for 3,2-HOPO chelator and 3,2-HOPO TOP-Succinimide at 0.25 µg mL^−1^ with ACC at 6 mg mL^−1^. Data reported as RSD (Area), n = 6.

	Six Different Injections from a Single Filtration	Six Different Injections from Six Independent Filtrations
**3,2-HOPO chelator**	2.34%	2.93%
**3,2-HOPO TOP-succinimide**	1.46%	3.01%

## Data Availability

Data is contained within the article and supplementary material.

## Supplementary Materials

The following supporting information can be downloaded at: https://www.mdpi.com/article/10.3390/molecules28062626/s1, Table S1: List of the columns tested in this study; Table S2: Analytical performance illustrating the screening results for separation of *N*-hydroxysuccinimide and succinimide obtained on eight different columns in isocratic mode with 0.1% TFA in water at 40 °C; Table S3: LOD and LOQ obtained for the 5 impurities spiked in an ACC sample (N-hydroxysuccinimide, succinimide, EDC-urea, 3,2-HOPO chelator, and 3,2-HOPO TOP-succinimide); Figure S1: Chromatograms obtained after the filtration protocol for ACC samples spiked with different concentrations of N-hydroxysuccinimide and succinimide and resulting calibration lines with triplicate injections; Figure S2: Chromatograms obtained after the filtration protocol for ACC samples spiked with different concentrations of EDC-urea and resulting calibration lines with triplicate injections.
