# Supplementary material for: Tackling Issues Observed during the Development of a Liquid Chromatography Method for Small Molecule Quantification in Antibody-Chelator Conjugate
Source: Molecules. 2023 Mar 14;28(6):2626. doi: 10.3390/molecules28062626 (PMC10055815; doi:10.3390/molecules28062626)
Supplement: Supplementary file 1 [file molecules-28-02626-s001.zip › molecules-2272035-supplementary.pdf]

Supplementary Material

Tackling Issues Observed during the Development of a Liquid Chromatography Method for Small Molecule Quantification in Antibody-Chelator Conjugate

Thomas Bouvarel [1,2], Nadine Bremeyer [3], Mimi Gao [3], Wiebke Holkenjans [3], Terence Hetzel [3], Reinhard Pell [3], Valentina D'Atri [1,2,*] and Davy Guillarme [1,2]

[1] School of Pharmaceutical Sciences, University of Geneva, CMU—Rue Michel Servet 1, 1211 Geneva, Switzerland
[2] Institute of Pharmaceutical Sciences of Western Switzerland, University of Geneva, CMU—Rue Michel Servet 1, 1211 Geneva, Switzerland
[3] Bayer AG, 42096 Wuppertal, Germany
* Correspondence: valentina.datri@unige.ch; Tel.: +41-22-379-33-58

Table S1. List of the columns tested in this study.

Provider	Name	Dimensions (mm)	Particle Size (µm)	Interaction Mechanism (Classified by Order of Importance)
Waters	CSH fluorophenyl	2.1 × 100	1.7	π –π, ionic, dipole-dipole, steric hindrance, hydrophobic
Phenomenex	Synergi Polar-RP	2.0 × 100	2.5	π –π, hydrophobic, dipole-dipole
Waters	Premier BEH C18 AX	2.1 × 100	1.7	Hydrophobic, ionic
Waters	Premier HSS T3	2.1 × 100	1.8	Hydrophobic, H-bonds, dipole-dipole
Phenomenex	Luna Omega polar	3.0 × 100	3.0	Hydrophobic, H-bonds, dipole-dipole
Thermo Scientific	Hypersil GOLD aQ	2.1 × 100	1.9	Hydrophobic, H-bonds, dipole-dipole
Agilent	Zorbax SB Aq	2.1 × 100	1.8	Hydrophobic, H-bonds, dipole-dipole
Phenomenex	Kinetex Polar	3.0 × 100	2.6	Hydrophobic, H-bonds, dipole-dipole

Table S2. Analytical performance illustrating the screening results for separation of *N*-hydroxysuccinimide and succinimide obtained on eight different columns in isocratic mode with 0.1% TFA in water at 40 °C.

Column	Retention Factor (N-hydroxy-succinimide)	Retention Factor (Succinimide)	Asymmetry (N-hydroxy-succinimide)	Asymmetry (Succinimide)	Resolution
CSH fluorophenyl	0.45	0.57	4.08	8.10	2.6
Synergi Polar-RP	1.02	1.30	1.12	1.23	2.5
Premier BEH C18 AX	0.71	1.15	2.62	4.37	3.7
Premier HSS T3	0.88	1.53	1.81	1.94	5.8
Luna Omega polar	0.76	1.31	1.37	1.47	6.6
Hypersil GOLD aQ	0.52	0.85	1.93	1.68	2.7
Zorbax SB Aq	0.56	1.01	1.51	1.88	4.8
Kinetex Polar	0.3	0.62	1.62	1.69	5.0

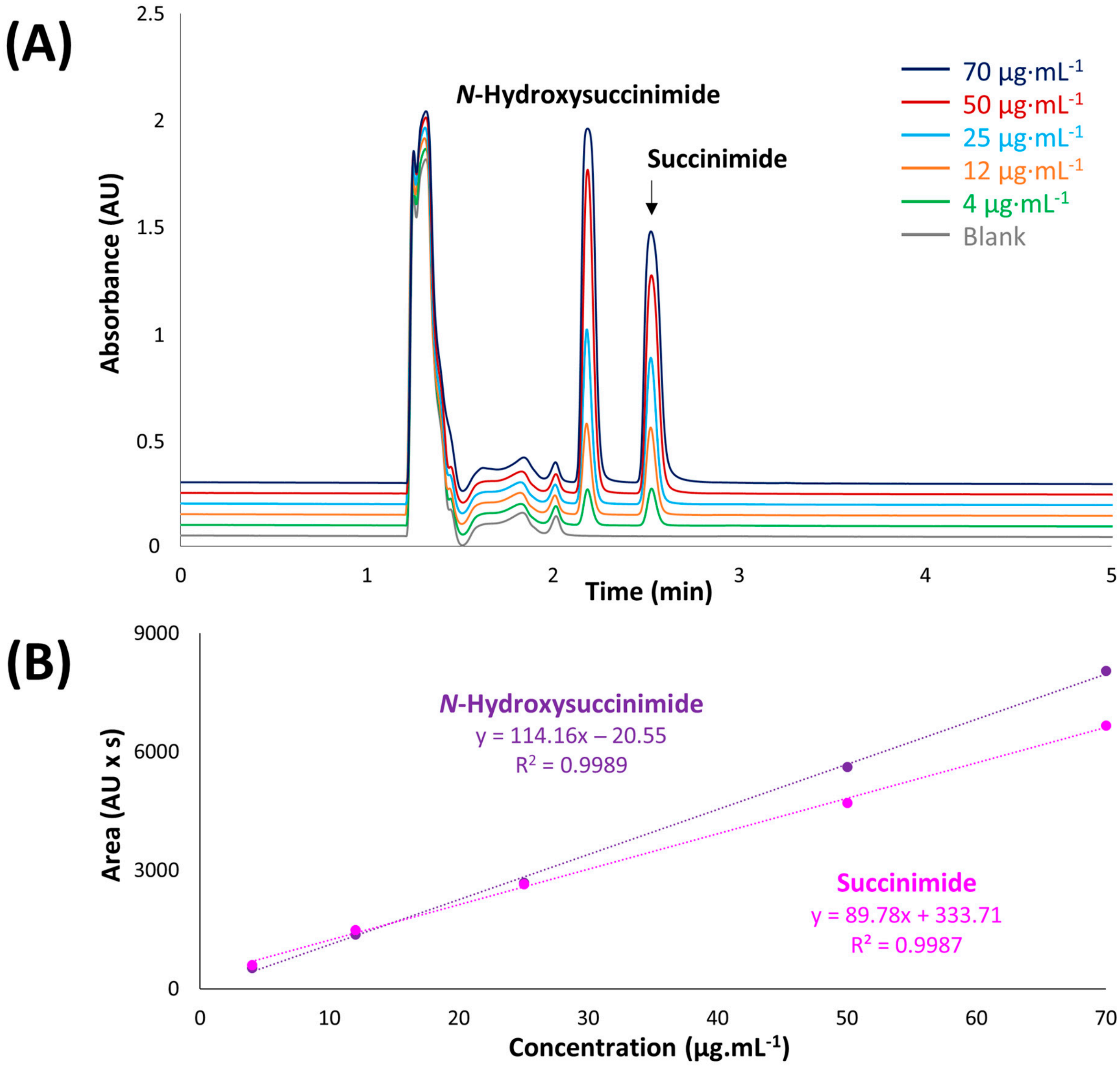

Figure S1. Chromatograms obtained after the filtration protocol for ACC samples (**A**) spiked with different concentrations of N-hydroxysuccinimide and succinimide and (**B**) resulting calibration lines with triplicate injections.

.

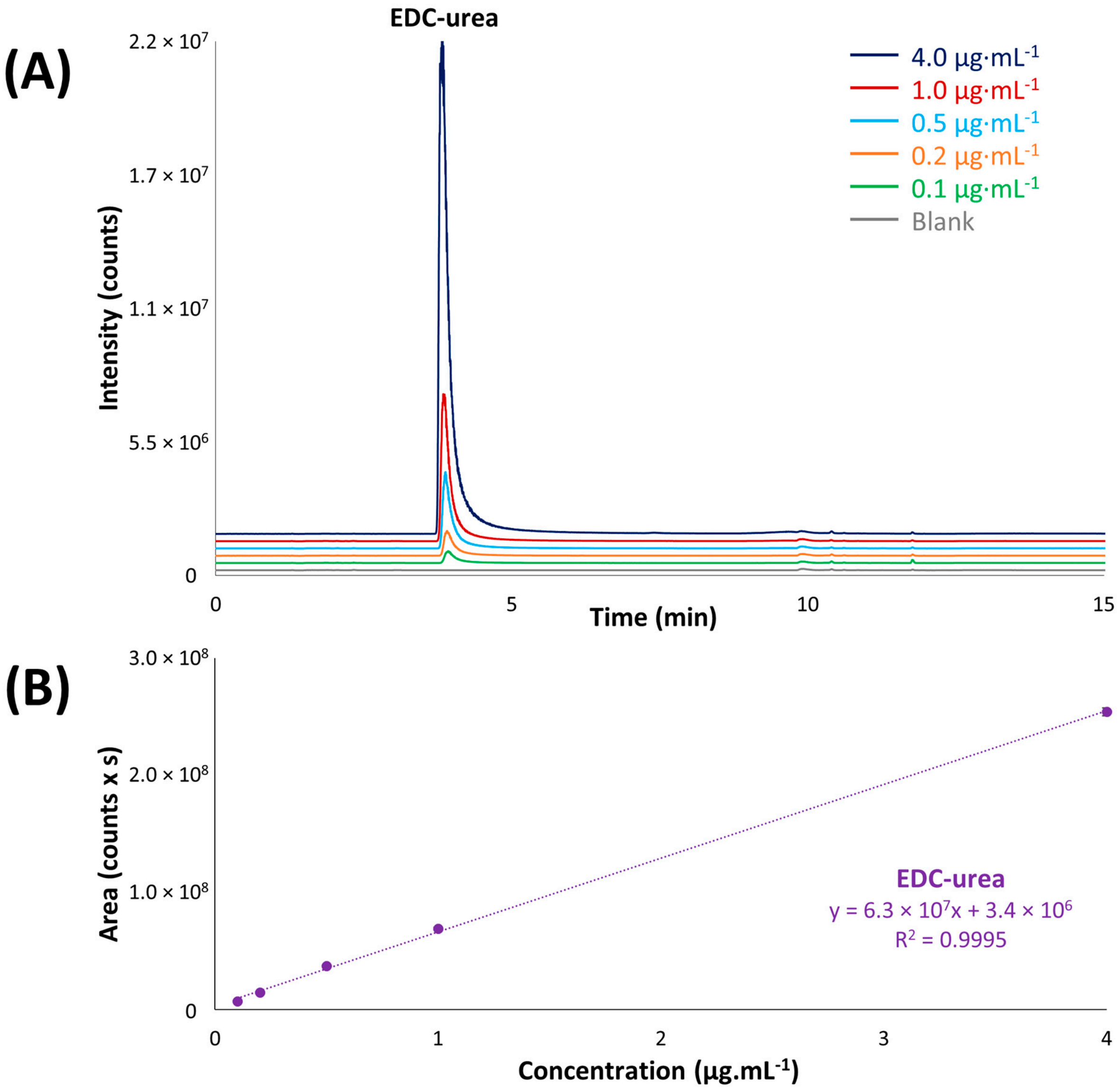

Figure S2. Chromatograms obtained after the filtration protocol for ACC samples (**A**) spiked with different concentrations of EDC-urea and (**B**) resulting calibration line with triplicate injections.

Table S3. LOD and LOQ obtained for the 5 impurities spiked in an ACC sample (N-hydroxysuccinimide, succinimide, EDC-urea, 3,2-HOPO chelator, and 3,2-HOPO TOP-succinimide).

	**LOD ($\mu g.mL^{-1}$)**	**LOQ ($\mu g.mL^{-1}$)**
***N*-hydroxysuccinimide**	0.024	0.080
**succinimide**	0.024	0.080
**EDC-urea**	0.0015	0.0050
**3,2-HOPO chelator**	0.015	0.050
**3,2-HOPO TOP-succinimide**	0.015	0.050
